# Preparation of Original and Calcined Layered Double Hydroxide as Low-Cost Adsorbents: The Role of the Trivalent Cation on Methylene Blue Adsorption

**DOI:** 10.3390/molecules28124717

**Published:** 2023-06-12

**Authors:** Raquel Trujillano, Vicente Rives, Rodrigo Miguel, Beatriz González

**Affiliations:** Departamento de Química Inorgánica, Universidad de Salamanca, 37008 Salamanca, Spain; rmiguemae@gmail.com (R.M.); bei@usal.es (B.G.)

**Keywords:** hydrotalcite, memory effect, adsorption, methylene blue

## Abstract

Layered double hydroxides with the hydrotalcite-like structure, containing Mg^2+^, Al^3+^, and Fe^3+^ (with different Al/Fe ratios) in the layers, have been synthesized and fully characterized, as have the mixed oxides formed upon their calcination at 500 °C. Both series of solids (original and calcined ones) have been tested for methylene blue adsorption. In the case of the Fe-containing sample, oxidation of methylene blue takes place simultaneously with adsorption. For the calcined samples, their reconstruction to the hydrotalcite-like structure plays an important role in their adsorption ability.

## 1. Introduction

Layered double hydroxides (LDHs) are synthetic or natural hydroxides with a lamellar structure, containing at least two types of different cations and an interlayer region occupied by anions and water molecules. The most abundant mineral with this type of structure is a magnesium and aluminum hydroxycarbonate whose formula corresponds to [Mg_6_Al_2_(OH)_16_]CO_3_·4H_2_O, hydrotalcite; so, these compounds are also known as hydrotalcites or hydrotalcite-like compounds [1,2,3]. The structure of a hydrotalcite is based on that of brucite Mg(OH)_2_, constituted by close hexagonal packing of hydroxyl groups with all octahedral holes occupied by Mg^2+^ cations every two interlayers; the octahedra share vertices and the sheets are linked to each other by hydrogen bonds. In hydrotalcite, some of the divalent cations are isomorphically replaced by cations of similar radii but greater charge, which produces an excess quantity of positive charge in the brucite-type sheets that is balanced by anions located in the interlaminar region together with water molecules, linked by hydrogen bonds [4,5,6]. The variety of compositions of these hydrotalcite-type compounds is very large, with a general formula: [M^2+^_1−x_ M^3+^_x_ (OH^−^)_2_]A^n−^_x/n_·mH_2_O [7,8]. The value of x corresponds to the so-called “metal molar fraction” of the trivalent cation, and its value usually ranges between 0.25 and 0.33. The substitution of the Mg^2+^, Al^3+^ cations and/or the interlaminar anion in hydrotalcite by other cations and anions lead to new isomorphic compounds, called hydrotalcite-type compounds, or simply hydrotalcites, modifying the properties of the original hydrotalcite [9]. The interlayer carbonate anion can also be easily substituted for other anions since it is bound to the framework by weak electrostatic forces; the only requirement is that the interlayer anion does not form complexes with the cations that form the hydrotalcite; these anions can be halides, oxyanions, polyoxoanions, oxometallates, polyoxometalates, anionic complexes, organic anions, biochemical anions, etc. [10,11,12].

One of the most important properties of this type of compound is the so-called “memory effect” [13]: the calcination of hydrotalcites at moderate temperatures (500–700 °C) produces mixed oxides able to reconstruct the original lamellar structure in the presence of anions dissolved in water [14].

The most widely used method to prepare hydrotalcites is coprecipitation [15], as it permits the production of large quantities of hydrotalcite in a short time by controlling parameters such as the concentration of metal salts, the type of reagents, the reaction temperature, the washing and drying conditions, or the pH, being very useful for the synthesis of hydrotalcites with carbonate as the interlayer anion [16,17].

These materials are of great interest due to their large number of applications, their low cost, ease of preparation, and the wide variety of compositions with which they can be synthesized.

They can be used as catalysts, catalyst precursors, composite fillers, or pollutant absorbers/adsorbents in water [18,19], for controlled drug release, as ceramic pigment precursors, as adsorbents, and as ion exchangers [13,20,21,22].

The aim of this research focuses on the absorption of methylene blue by hydrotalcites or calcined hydrotalcites because it is one of the basic dyes in the textile industry [23] and whose retention has been widely studied as an indicator of the performance of absorption by various materials such as zeolites [24], Mg-Fe or Mg-Al hydrotalcites [25], or Zn-Al hydrotalcites [26]. To assess the incidence of the Fe^3+^ cation in the adsorption process, the synthesis of three lamellar solids with Mg, Al, or Fe in the layers and carbonate as interlamellar anion has been carried out. The original solids have been calcined, and all the products obtained have been widely characterized and tested for methylene blue absorption.

Layered double hydroxides (LDHs) are synthetic or natural hydroxides with a lamellar structure, containing at least two types of different cations and an interlayer region occupied by anions and water molecules. The most abundant mineral with this type of structure is a magnesium and aluminum hydroxycarbonate whose formula corresponds to [Mg_6_Al_2_(OH)_16_]CO_3_·4H_2_O, hydrotalcite; so, these compounds are also known as hydrotalcites or hydrotalcite-like compounds [1,2,3]. The structure of hydrotalcite is based on that of brucite Mg(OH)_2_, constituted by close hexagonal packing of hydroxyl groups with all octahedral holes occupied by Mg^2+^ cations every two interlayers; the octahedra share vertices and the sheets are linked to each other by hydrogen bonds. In hydrotalcite, some of the divalent cations are isomorphically replaced by cations of similar radii but greater charge, which produces an excess of positive charge in the brucite-type sheets that is balanced by anions located in the interlaminar region together with water molecules, linked by hydrogen bonds [4,5,6]. The variety of compositions of these hydrotalcite-type compounds is very large, with a general formula: [M^2+^_1−x_ M^3+^_x_ (OH^−^)_2_]A^n−^_x/n_·mH_2_O [7,8]. The value of x corresponds to the so-called “metal molar fraction” of the trivalent cation, and its value usually ranges between 0.25 and 0.33. The substitution of the Mg^2+^, Al^3+^ cations and/or the interlaminar anion in hydrotalcite by other cations and anions lead to new isomorphic compounds, called hydrotalcite-type compounds, or simply hydrotalcites, modifying the properties of the original hydrotalcite [9]. The interlayer carbonate anion can also be easily substituted for other anions since it is bound to the framework by weak electrostatic forces; the only requirement is that the interlayer anion does not form complexes with the cations that form hydrotalcite; these anions can be halides, oxyanions, polyoxoanions, oxometallates, polyoxometalates, anionic complexes, organic anions, biochemical anions, etc. [10,11,12].

Calcination of a hydrotalcite at moderate temperatures produces mixed oxides. One of their most important properties of them is the so-called “memory effect” [13]; this effect consists of the reconstruction of the layered structure after calcination of hydrotalcites at moderate temperatures (500–700 °C); recovery of the lamellar structure is achieved when the calcined solids are dispersed in a water solution containing the desired anion [14]. When calcination has been carried out at elevated temperatures, the oxides obtained have a very stable structure, such as the spinel structure, and reconstruction of the layered structure is rather difficult, if not impossible.

The most widely used method to prepare hydrotalcites is coprecipitation [15]. This procedure consists of the slow addition of a salts aqueous solution with the desired cations to another salt solution with the anion chosen. The pH value selected during the mixing process must be the optimal one to allow the simultaneous precipitation of the double hydroxides (coprecipitation). This method permits the production of large quantities of hydrotalcite in an easy way and a short time by controlling parameters such as the concentration of metal salts, the type of reagents, the reaction temperature, the washing and drying conditions, or the pH, being very useful for the synthesis of hydrotalcites with carbonate as the interlayer anion [16,17]. These are some of the reasons that justify the choice of this method in this scientific work.

These materials are of great interest in environmental as well as industrial applications due to their low cost, ease of preparation, and the wide variety of compositions with which they can be synthesized [16]. These solids are suitable as catalysts, catalyst precursors, composite fillers, or pollutant absorbers/adsorbents in water [18,19], for controlled drug release, as ceramic pigment precursors, as adsorbents, and as ion exchangers [13,20,21,22].

The aim of this research work focuses on the absorption of methylene blue by hydrotalcites or calcined hydrotalcites because it is one of the basic dyes in the textile industry [23], and whose retention has been widely studied as an indicator of the performance of absorption by various materials such as zeolites [24], Mg-Fe or Mg-Al hydrotalcites [25], or Zn-Al hydrotalcites [26]. No studies about the effect in the adsorption capacity or procedure of the substitution of a part of Al^3+^ by Fe^3+^ in the Mg-Fe or Mg-Al hydrotalcites have been reported previously. This study intends to answer this question, and so, to assess the incidence of the Fe^3+^ cation in the adsorption process; the synthesis of three lamellar solids with Mg, Al, or Fe in their layers and carbonate as their interlamellar anion has been carried out. The original solids have been calcined, and all the products obtained have been widely characterized and tested for methylene blue absorption.

## 2. Results and Discussion

### 2.1. Characterization

The results of the element chemical analysis (ECA) given as mass percentages of Mg, Fe, and Al are included in Table 1. Only the original samples have been submitted to this analysis since the calcination process removes CO_2_ and H_2_O, but not the cations, leading to a mixture of oxides. Since powder X-ray diffraction does not identify mixed oxide phases, the compositions of the calcined samples are expressed as mixtures of simple oxides.

The formulae have been determined considering the general chemical formula of the hydrotalcite-type compounds [27] and the results of element chemical analysis for cations. From the FT-IR spectroscopy data (in the following section), it has been confirmed that carbonate is the only interlayer anion, so the amount of the anion is needed to balance the positive charge of the layer [16]. The number of water molecules is calculated from the results of the TG analysis [18]. The molecular formulas of the solids obtained after calcination are also included in Table 1. Their formulas have been calculated, bearing in mind that calcination gives rise to a mixture of simple oxides in which the cationic molar ratio is maintained. The M^2+^/M^3+^ molar ratio in the original solids is close to 2:1, as in the synthesis mixture, which suggests that there has been complete precipitation; a significant deviation is only observed for the Mg/Fe sample. The molar cation ratios found for the synthesized samples coincide with that of hydrotalcite-type compounds; therefore, we can assume that the solids obtained have that structure [28].

**Figure 1:**
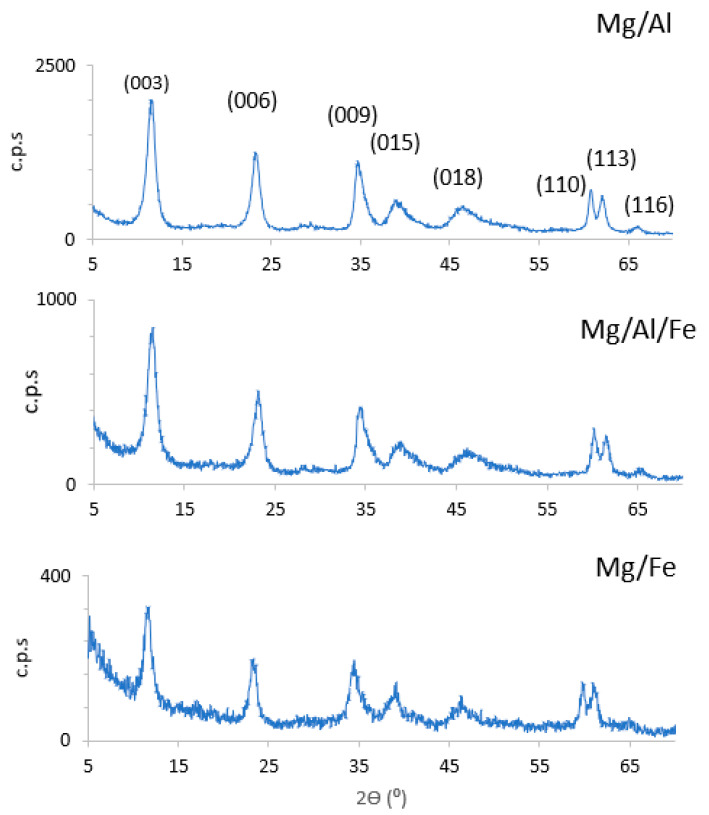
PXRD diagrams of the original samples.

The powder X-ray diffractograms (PXRD) of the original samples (Figure 1) show intense peaks whose positions and relative intensities correspond to laminar materials with a hydrotalcite-like structure [29]. The positions of these peaks coincide with those of similar solids with a hydrotalcite-like structure with carbonate as their interlayer anion [30].

The most characteristic peaks of this kind of layered hydroxycarbonates are the very sharp peaks of decreasing intensity corresponding to the basal plane diffractions (00l). The first one is related to the (003) diffraction planes and their spacing value, calculated using the Bragg equation, and corresponds to the height of the interlayer space plus the thickness of a brucite-type layer. The spacings of the diffractions by the (006) and (009) planes are, respectively, one-half and one-third of those corresponding to the (003) planes. The spacing value for diffraction (003) allows the crystal parameter c to be determined. In its turn, the position of the (110) plane diffraction is also very important since its spacing corresponds to one-half of the lattice parameter a [31].

Table 2 includes the positions of the diffraction peaks recorded in the diffractograms in values of 2Ɵ (°) and spacing (d/Å), as well as the Miller indices (hkl) of the diffraction planes to which they correspond.

These crystallographic parameters a and c included in Table 2 are very close to those reported in the literature for hydrotalcite-like solids [16]. The crystallographic parameter *c* for a natural hydrotalcite is 23.52 Å, three times the spacing of the (003) planes, although to determine it more precisely, the positions of the peaks of the (003) and (006) planes are sometimes used in the following equation:

*c* = 3 × 1/2 × [d(003) + 2d(006)]

The crystallographic parameter *a* (average cation-cation distance within a sheet) has been calculated using the equation:

*a* = 2d (110)

The full width at half maximum (FWHM) is related to the size of the crystal in the direction perpendicular to the diffraction plane. The size of the crystallite calculated in the *c* direction by using the Scherrer equation and considering the FWHM value of the diffraction peak due to the (003) planes is also included in Table 3. As the crystal size increases, the peak becomes narrower. In view of the diffraction peaks related to the 003 planes, the crystallinity increases in the trend: Mg/Al < Mg/Al/Fe < Mg/Fe.

**Figure 2:**
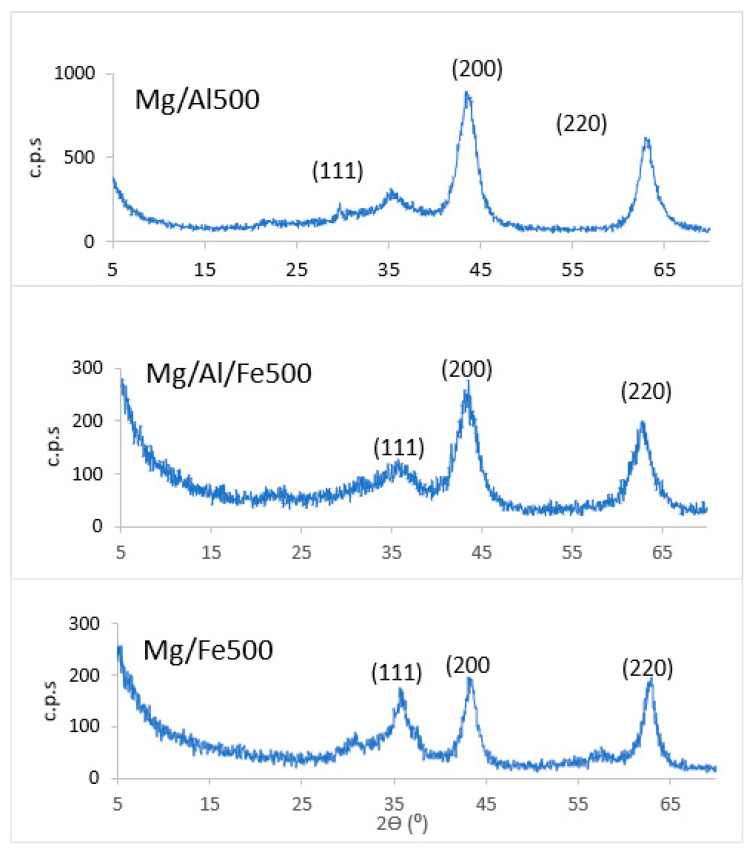
PXRD diagrams of the calcined samples.

The PXRD diagrams of the calcined samples (Figure 2) show diffraction peaks whose positions match with those reported in the literature [32] for MgO periclase confirming that at this calcination temperature only MgO is formed as a crystalline phase; the other cations must be forming amorphous species.

The peaks recorded for the calcined samples correspond to diffraction by the (111), (200), and (220) planes of MgO, as expected for a face-centered cubic structure. In any case, in the case of the Mg/Fe500 sample, a rather poorly defined and not very intense peak is observed close to a value of 30°, which may be due to the incipient crystallization of a spinel-type phase, MgFe_2_O_4_.

Table 4 summarizes the positions of the diffraction peaks recorded in the diffractograms, as well as the Miller indices (hkl) of the diffraction planes to which they correspond. Since MgO has a cubic structure, the value of the parameter a has been calculated according to the equation a^2^ = (h^2^ + k^2^ + l^2^)d^2^ [33]. The crystallite size values (*D*), calculated according to the Scherrer equation, are also included. Both parameters *a* and *D* are included in Table 4.

**Figure 3:**
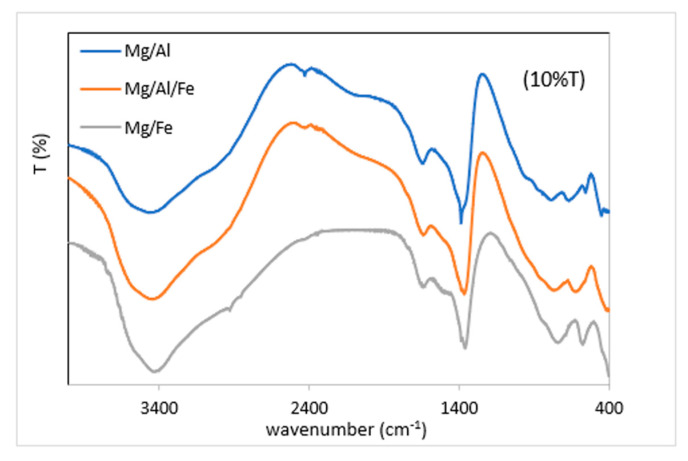
FT-IR spectra of the original samples.

The bands recorded in the FT-IR spectra of the original samples, Figure 3, are typical of hydrotalcite-like solids [35]. The slight shift in their positions is due to the different compositions of the samples. Table 5 includes the positions of the recorded bands, as well as the vibrational modes to which they are assigned.

The band close to 3500 cm^−1^ corresponds to the O-H stretching vibration of the water molecules and of the OH groups in the sheets. Its position varies depending on the M^2+^/M^3+^ ratio and the nature of the metals. A shoulder is observed at 3100 cm^−1^ due to the O-H stretching mode of hydroxyl groups hydrogen-bonded to the interlayer carbonate anions. The band due to the H-O-H angular bending vibration of interlaminar water is observed around 1600 cm^−1^. The positions and number of bands of the carbonate ion depend on its symmetry. The free carbonate ion has a D_3h_ symmetry, and thus, three normal vibrational modes are expected in the infrared spectrum as three bands at 1380–1350 cm^−1^, 880–850 cm^−1^ and 690–660 cm^−1^. When located between two brucite-type sheets, a decrease in C_2v_ symmetry can occur, activating the band between 1100–1000 cm^−1^ that is recorded in the spectra of the Mg/Al and Mg/Fe samples and very weakly in that of Mg/Al/Fe. The remaining bands are due to the vibrational and translational modes of the metal cations and the hydroxyl groups of the brucite sheets [35,36]. Finally, the band at 750 cm^−^^1^ corresponds to the metal-OH vibration, whose position also depends on the type of metal.

**Figure 4:**
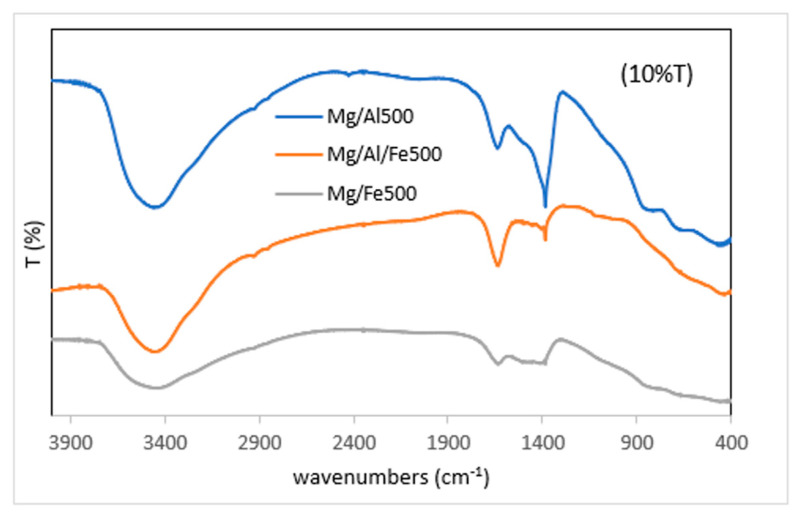
FT-IR spectra of the calcined samples.

Bands due to the metal-hydroxide vibrational modes are no longer recorded in the spectra of the calcined samples (Figure 4 and Table 5), and some weak bands attributable to metal-oxygen vibrational modes are recorded. These features confirm the loss of the layered structure. Due to the willingness of these samples to adsorb water or CO_2_, weak bands attributable to H_2_O or carbonate vibrational modes are recorded.

**Figure 5:**
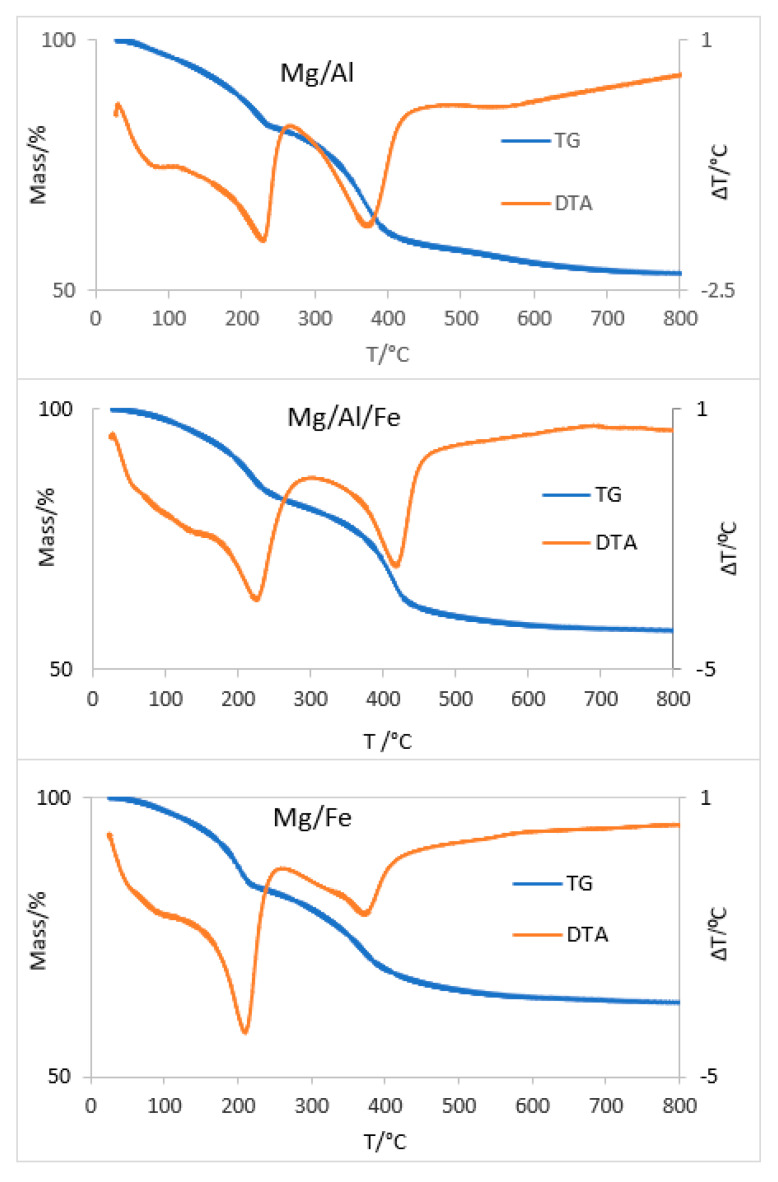
TG (left scale) and DTA (right scale) curves of the original samples.

The TG and DTA curves are recorded for the original samples and are included in Figure 5; they are characteristics of lamellar solids with a hydrotalcite-like structure [37].

The data obtained from the TG and DTA analyses have been used both to calculate the amount of crystallization water in the samples and to select the calcination temperature, to obtain the desired oxides. The curves of the three original samples are similar to each other; the mass losses are attributable to the same effects. The DTA curve of the Mg/Al sample shows three endothermic processes with minima close to 106, 229, and 370 °C and a weak shoulder at ca. 550 °C. The first two thermal effects are overlapped and are accompanied by a total mass loss close to 18%; they correspond to the removal of surface and interlaminar water, respectively. The third endothermic process is due to the removal of CO_2_ and H_2_O from the interlamellar anion and the hydroxide groups that form the layers, with a mass loss of 22%. The final mass loss (6%) should be due to the removal of residual hydroxide groups, and it coincides with the temperature range of the fourth endothermal shoulder. The total mass loss for this sample is around 46%. The MgFe sample shows two main mass losses; the first one, approximately 18%, is recorded between 25 and 260 °C. In this range, the DTA curve shows two overlapping endothermic processes with minima around 100 °C and 210 °C, corresponding to the removal of physisorbed and interlaminar water loss, respectively. The third endothermic effect of the DTA is centered near 375 °C, with a 20% mass loss in the TG curve. In this case, CO_2_ is released from interlayer carbonate and water vapor from the layered OH groups, causing the destruction of the layered structure. This process corresponds to a loss of mass of 38%. From the end of this last thermal effect and until the end of the analysis, a small mass loss of about 1% is observed, which could be due to loss of water or strongly retained CO_2_. Similar values are found for Mg/Fe/Al.

**Figure 6:**
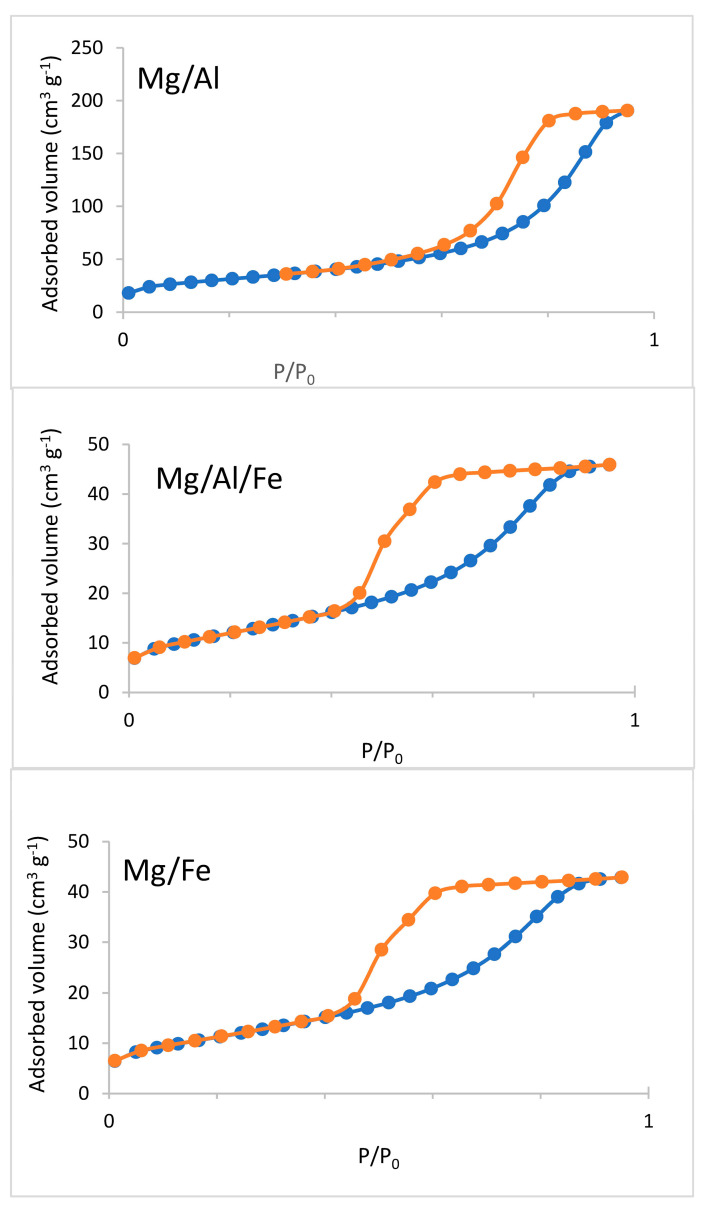
N_2_ adsorption-desorption isotherms of the original samples. Adsorption branch in blue and desorption branch in orange.

**Figure 7:**
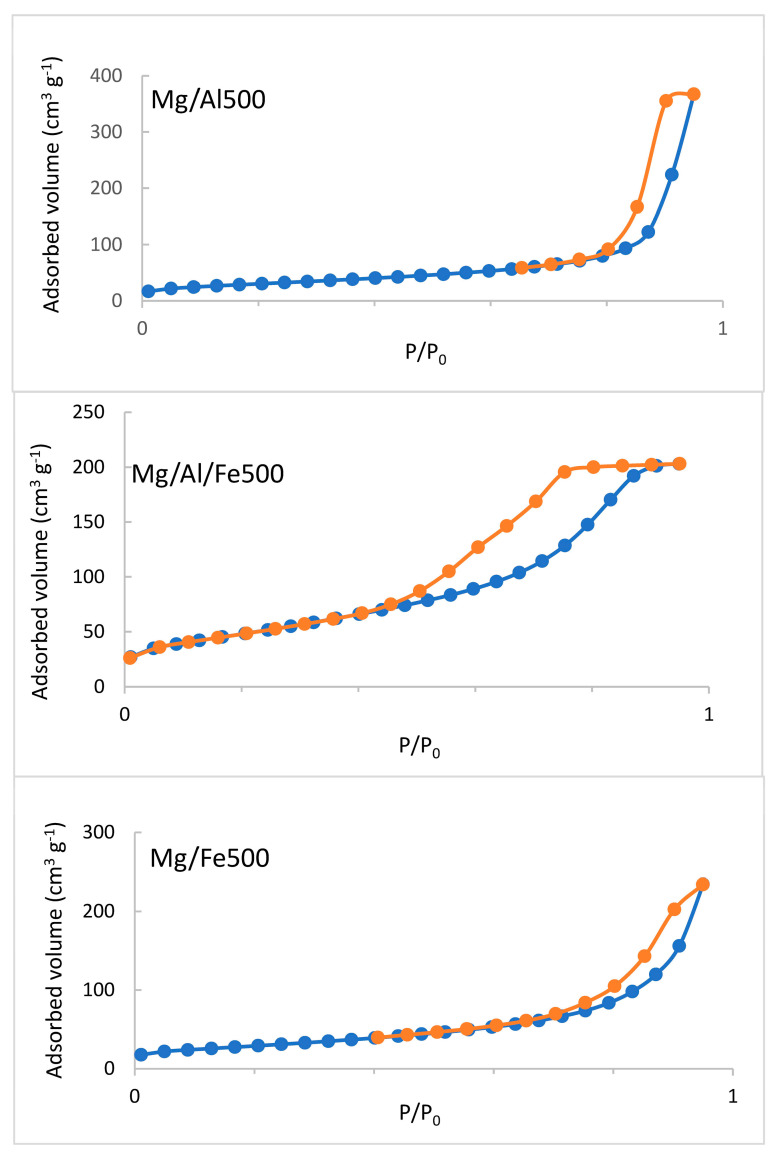
N_2_ adsorption-desorption isotherms of the calcined samples. Adsorption branch in blue and desorption branch in orange.

The specific surface area has been assessed by means of N_2_ adsorption-desorption isotherms that are included in Figure 6 and Figure 7. The adsorption isotherms of the original samples (Figure 6) belong to type IV of the IUPAC classification [38]. This kind of isotherm is typical of mesoporous samples; all of them show an H2-type hysteresis loop according to the IUPAC classification [39]. This type of loop corresponds to samples with inkbottle pore shapes. The shape of the Mg/Al500 and Mg/Al/Fe500 samples adsorption-desorption isotherms (Figure 7) are Type IV, rather similar to those of Mg/Al and Mg/Al/Fe solids. After calcination, Mg/Fe/Al500 and Mg/Fe500 solids show a higher specific surface area than the original samples, but the Mg/Al500 sample has the same S_BET_ value as the Mg/Al solid. The hysteresis loop of Mg/Al500 is narrower than that of its original sample, indicating a better definition of the pores’ shape due to the calcination. On the other hand, the Mg/Fe500 sample isotherm (Figure 7) shows an H1-type hysteresis loop revealing that the pore shapes of the original samples have evolved to a better-defined cylindrical-like pore channel after calcination [38].

Table 5 summarizes the S_BET_ (specific surface area calculated by the BET method), and S_t_ (external surface area) values together with the regression coefficients from the calculations carried out. Both are rather similar; it can be said that micropores are not present in any of the obtained solids.

### 2.2. Adsorption of Methylene Blue (MB)

**Figure 8:**
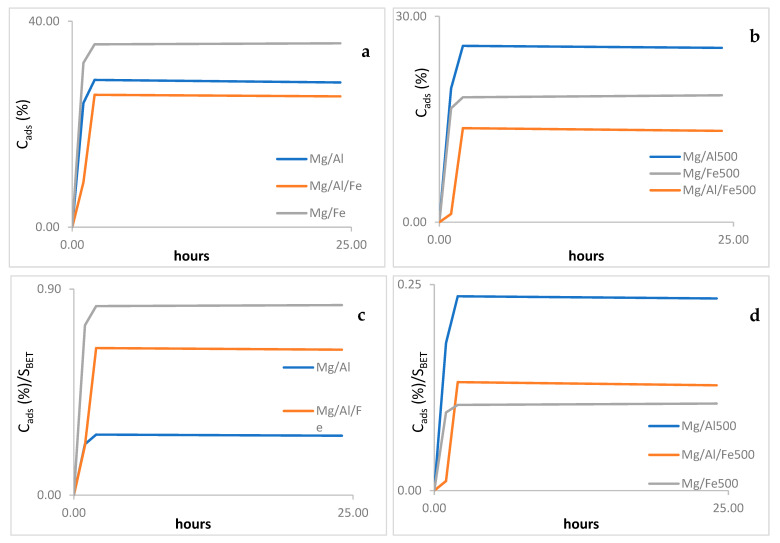
Adsorption results. (**a**,**c**) graphics show C_ads_ against time, and (**b**,**d**) represents C_ads_/S_BET_ against time.

Figure 8 shows the percentage of MB adsorbed (C_ads_) and also C_ads_/S_BET_ against time for the original and the calcined samples. The C_ads_ values have been calculated by using the following equation: C_ads_ = 100 − (A/A_0_)%, where A and A_0_ are the band intensities of the band recorded at 660 nm between any time during the reaction and the initial one (zero time reaction), respectively.

The amount of MB adsorbed (C_ads_) increases quickly during the first contact when using Mg/Al and Mg/Fe and somewhat more slowly when Mg/Al/Fe is the adsorbent (Figure 8a), the k_1_ values (Table 6) confirm the lower adsorption rate of these samples.

Then the adsorption rate decreases and remains almost constant from two to twenty-four contact hours. Only a slight increase is observed for the Mg/Al sample. Plotting C_ads_ against the contact time, it is evident that Mg/Fe has a higher adsorption capacity, while those of the other two samples are similar to each other and lower. These results do not evidence if the substitution of Al^3+^ for Fe^3+^ is the main responsible factor for the different results. So, to assess the adsorption capacity per unit surface area, C_ads_/S_BET_ has been plotted against contact time (Figure 8b), and it is evidenced that the highest adsorbent capacity is shown by the Mg/Fe sample and the lowest one by the Mg/Al sample. It seems very probable that the factors with the greatest impact on the MB adsorption capacity of these samples are the nature of the trivalent cation and their molar cation ratio. The higher adsorption capacity per surface area unit of the Mg/Fe sample may also be due to its oxidizing ability, thus adding its oxidizing capacity to its adsorbent capacity, given its higher Fe^3+^ content as the only trivalent cation.

In order to study the mechanism that controls the MB adsorption on the adsorbents, the two most widely used models have been used, namely, pseudo-first-order (Equation (1)) and pseudo-second-order (Equation (2)) [40,41,42].

q_t_ = q_e_ [1 − e(−k_1_t)] (1)

q_t_ = k_2_q_e_^2^t/(1 + k_2_q_e_t) (2)

The kinetic parameters (Table 6) demonstrate that pseudo-second-order parameters, with a low correlation coefficient, are senseless for original and calcined Mg/Fe/Al samples and do not fit the experimental data for the other samples, thus confirming that chemical adsorption is not the predominating type in this experiment. The adsorption of MB can better be described by the pseudo-first-order equation because it fits the experimental data better (Table 6), confirming that the process was controlled by physical adsorption.

Even though the conditions of the experiment were not probably the most suitable conditions for this type of reaction to take place, it is possible that the degradation of the MB took place to a greater extent than its adsorption. Several papers explain the role of Fe^3+^ species in the enhancement of MB photodegradation; Balu et al. [43] explained the enhancement of the degradation of MB under visible light by nanocomposites with different amounts of SiO_2_-Fe_2_O_3_ because the Fe_2_O_3_ nanoparticles used to trap electrons enhance the photocatalytic activity. Waghchaure et al. [44] proved that Fe^3+^ addition to ZnO enhanced the photocatalytic performance in the degradation of MB because the decrease of the band gap facilitates the electron transfer from the valence band to the conduction band, showing that their combination with water and oxygen generates reactive species. In brief, the better degradation efficiency due to the Fe^3+^ species is primarily ascribed to the role of this cation as an electron-transfer mediator [45].

The study of the adsorbent capacity of the calcined samples was carried out in the same way as for the original samples; as can be observed in Figure 8c, the adsorption rate in the first hour is higher for samples Mg/Al500 and Mg/Fe500. The adsorption rate pattern in these samples is like that already observed for the original samples. In this case, the highest percentage of adsorbed MB is shown by the Mg/Al500 sample, even though it displays a lower S_BET_ than the Mg/Fe sample.

On plotting C_ads_/S_BET_ against the contact time (Figure 8d), it is clear that in the calcined samples, the highest adsorbent capacity is observed for sample Mg/Al500. It can also be observed that the increase in contact time after the initial two hours is not a parameter that significantly affects the adsorbing capacity for any solid.

According to the PXRD results for the calcined samples, the claim that the trivalent cation forms an amorphous phase on which the crystalline MgO is supported. However, in the diffractogram of the Mg/Fe500 sample, a rather poorly defined and not very intense peak was observed, close to a value of 30°, probably due to the beginning of the crystallization of a very stable spinel-type oxide.

Due to the memory effect of hydrotalcites, these solids, after being calcined, can reconstruct the lamellar structure if the calcination temperature has not given rise to the formation of stable mixed oxides such as spinels. But, for samples containing transition cations such as Fe^3+^, the temperature at which the spinel phase begins to form is lower than for Mg-Al hydrotalcites, probably due to additional stabilization, because of the crystal field effect of the spinel phase containing transition metal cations [46,47]. For this reason, and despite the larger specific surface area of the Mg/Al/Fe500 and Mg/Fe500 samples, the incipient formation of spinels prevents reconstruction to the same extent as in the Mg/Al500 sample. Thus, in this sample, although with a lower specific surface area than that of Mg/Fe500 and like that of Mg/Al/Fe500, two effects are added that give rise to a greater decrease in the concentration of contaminants in the initial solution, namely, adsorption and intercalation. The first is a surface phenomenon, and the second is probably due to the incorporation of the MB molecule into the interlaminar space during the reconstruction process.

## 3. Materials and Methods

To synthesize the samples, Na_2_CO_3_, NaOH, Al(NO_3_)_3_·9H_2_O, Mg(NO_3_)_2_·6H_2_O, and Fe(NO_3_)_3_·9H_2_O were used, as well as KBr for FT-IR measurements; all chemicals were from Panreac. The gases used for textural and thermal analyses as well as the liquid nitrogen were supplied by L’Air Liquide España, S. A.

The element chemical analysis (ECA) has been performed at Servicio General de Análisis Químico from the Universidad de Salamanca in AGILENT 7800 equipment (Murcia, Spain).

The powder X-ray diffractograms were recorded in a SIEMENS D5000 X-ray diffractometer with filtered Cu Kα radiation (λ = 1.5418 Å); the recording 2θ range was 5–75°, the current intensity and voltage conditions 30 mA and 40 kV, respectively, while the step and time were 0.05° every 1.5 s, thus resulting in a scanning speed of 2°/min. The FT-IR spectra were recorded in PERKIN-ELMER SPECTRUM-TWO (Madrid, Spain) equipment, averaging 12 scans with a nominal resolution of 4 cm^−1^ in the 4000 to 400 cm^−1^ range; the samples were prepared by the KBr pellet method. A PERKIN-ELMER LAMBDA 35 spectrophotometer has been used to collect the Visible-ultraviolet spectra between 1100 and 300 nm with an optical aperture of 2 nm and distilled water as a reference. Thermogravimetric and Differential Thermal analyses (TG-DTA) were carried out simultaneously in an SDT Q 600. Measurements were performed using a dynamic oxygen atmosphere of 50 L/min with a heating rate of 10 °C/min from room temperature to 1000 °C and using α-Al_2_O_3_ as a reference. A GEMINI VII 1390 analyzer was used for the determination of the specific surface area and the porosity of the samples from the adsorption/desorption isotherms of nitrogen recorded at −196 °C. Previous to the analysis, a given amount of a sample (100–200 mg) was heated at 110 °C under a stream of N_2_ for two hours in a Micromeritics FlowPrep 060 Sample Degass System to remove softly adsorbed species. The specific surface area has been calculated using the BET method, S_BET_ [38] and the external surface area, S_t_, was found following the t-plot method [39].

The samples have been prepared following the coprecipitation method [16]; for this purpose, 100 mL of an aqueous solution of the salts of the cations in the desired molar ratio to be incorporated into the solid have been added dropwise with continuous stirring to 100 mL of a solution of Na_2_CO_3_ at a pH previously adjusted to 10 by adding 1M NaOH. That pH was kept constant by adding the NaOH solution during mixing. The three syntheses started with 20 g of Mg(NO_3_)_2_·6H_2_O (0.078 moles), and the amounts of Al (NO_3_)_3_·9H_2_O and Fe (NO_3_)_3_·9H_2_O were calculated in order to obtain in each sample a molar cation ratio M^2+^/M^3+^ equal to two and an Al^3+^/Fe^3+^ ratio equal to one. The obtained solids were labeled as Mg/Al, Mg/Fe, and Mg/Al/Fe.

During the addition of the precursor solution to the Na_2_CO_3_ solution, a white (in the case of the Mg/Al sample) or brown (for the Mg/Fe and Mg/Al/Fe samples) precipitate was formed. When the addition was finished, the obtained suspension was submitted to centrifugation, and the solid was washed with distilled water until the removal of nitrates (from the starting salts) was complete. The absence of nitrates was verified by a qualitative analysis test using Mohr’s salt and concentrated sulfuric acid [48]. Once the absence of nitrate was confirmed, the solid was dried in an oven at 45 °C for two days. Approximately 3 g of the samples, as synthesized, were calcined at 500 °C in an oxidizing and static atmosphere for three hours. The solids thus obtained were labeled as Mg/Al500, Mg/ Fe500, and Mg/Al/Fe500.

The adsorption study, both for the original samples and the calcined ones, has been carried out by dispersing 100 mg of adsorbent in 100 mL of a 10 ppm MB aqueous solution. Then, the suspension formed was kept at room temperature and constant agitation for 24 h. Aliquots have been taken at different times to record its Vis-UV spectrum and to assess the percentage of contaminant adsorbed by the solids at each time by comparison with the absorbance of the initial solution. The visible-ultraviolet spectrum (Vis-UV) of the initial solution and of each of the aliquots collected after the preset contact time with the solids has been recorded between 1100 and 180 nm. Four bands are observed in the spectrum of the MB aqueous solution, two in the ultraviolet zone of the spectrum, at approximately 243 and 289 nm, and another two in the visible zone, at 607 and 660 nm. The maximum absorbance is shown by the band at 660 nm, and so it has been used to monitor the MB concentration.

## 4. Conclusions

The method used for the synthesis of the three hydrotalcites has been successful; the ECA results and the PXRD diagrams confirm that both the formulas of the solids obtained and their structures correspond to hydrotalcite-type laminar compounds. The FT-IR spectra results confirmed that carbonate is the only interlaminar anion, and the results of the N_2_ adsorption-desorption isotherms reveal that they are, as expected, mesoporous solids. These hydrotalcites evolve into a mixture of oxides after calcination at 500 °C.

The pseudo-first-order model fits the experimental data, thus confirming that physisorption controls the adsorption of the MB onto the adsorbent. The adsorption ability of the hydrotalcites and the oxides have been measured using Methylene Blue as a probe molecule. For all the samples, the adsorption equilibrium is reached after two hours. Regarding hydrotalcites, the highest efficiency of the sample Mg/Fe, the sample containing only Fe^3+^ as a trivalent cation, is probably due to the adsorbent capacity corresponding to its S_BET_ combined with the oxidizing capacity of the Fe^3+^ cation. The calcined samples, despite having a higher S_BET_ value, show a lower adsorption capacity than the original samples; this decrease can be attributed to the difference in structure. On the other hand, the best adsorbent is the Mg/Al500 sample, tentatively due to the reconstruction capacity that these samples have, attributable to the memory effect, which is superior in Mg/Al500, the solid that does not contain Fe^3+^ species.

Future experiments will focus on the design of MgFeAl with the same composition as the samples described here but with different S_BET_ values. These will be obtained by modulating the synthesis and post-synthesis parameters. The adsorption capacity of these solids and those derived from their calcination will be compared with the present results in order to implement a synthesis method to obtain ecomaterials suitable for minimizing the pollutant concentration in waters.

## Figures and Tables

**Table 1:** ECA results of the original samples and formulae of all solids.

Sample	%Mg *	%Al *	%Fe *	Mg/M^3+^ **	Al/Fe **	Formula
Mg/Al	18.97	10.36		2.03		[Mg_0.67_Al_0.33_(OH)_2_](CO_3_)_0.16_·0.84H_2_O
Mg/Al/Fe	19.24	5.75	10.31	1.99	1.15	[Mg_0.67_Al_0.18_Fe_0.16_(OH)_2_](CO_3_)_0.17_·0.78H_2_O
Mg/Fe	16.23		25.91	1.44		[Mg_0.59_Fe_0.41_(OH)_2_](CO_3_)_0.20_·0.89H_2_O
Mg/Al500						(MgO)_0.67_ + (Al_2_O_3_)_0.16_
Mg/Al/Fe500						(MgO)_0.67_ + (Al_2_O_3_)_0.09_ + (Fe_2_O_3_)_0.08_
Mg/Fe500						(MgO)_0.59_ + (Fe_2_O_3_)_0.20_

* mass %; ** molar ratio.

**Table 2:** Diffraction peak positions.

JCPDS 22-700 *	Mg/Al		Mg/Al/Fe		Mg/Fe		h k l
d/Å	2θ/°	d/Å	2θ/°	d/Å	2θ/°	d/Å	
7.841	11.49	7.68	11.57	7.63	11.63	7.63	0 0 3
3.898	23.19	3.83	22.3	3.82	23.2	3.81	0 0 6
2.599	34.94	2.58	34.41	2.59	34.57	2.60	0 0 9
2.329	38.88	2.31	39.12	2.29	38.82	2.30	0 1 5
1.959	46.57	1.95	46.18	1.93	46.18	1.95	0 1 8
1.540	59.9	1.52	59.060	1.53	60.3	1.55	1 1 0
1.497	60.8	1.49	60.8	1.50	61.2	1.52	1 1 3
1.432	66.2	1.40	65.75	1.41	65.0	1.43	1 1 6

* reference values, standard 22-700—JCPDS.

**Table 3:** Cell parameters c and a and crystallite size *D* values (Å) of the samples.

Parameter	Mg/Al	Mg/Al/Fe	Mg/Fe
***c***	23.01	22.09	22.87
***a***	3.04	3.08	3.1
***D***	83	85	98

**Table 4:** Positions of the main diffraction peaks of the calcined samples in 2θ (°) and d (Å). Miller indexes of the diffraction planes and *D* values are also included.

	MgO *	Mg/Al500		Mg/Al/Fe500		Mg/Fe500		h k l
	d/Å	2θ/°	d/Å	2θ/°	d/Å	2θ/°	d/Å	
	2.432	35.55	2.523	35.90	2.499	36.05	2.489	1 1 0
	2.107	43.45	2.0081	43.10	2.097	43.30	2.088	2 0 0
	1.490	63.2	1.470	62.90	1.476	62.65	1.482	2 2 0
*a*	4.213		4.233		4.213		4.233	
*D*	-	47			91			88

* Ref. [34].

**Table 5:** SBET and St values (m^2^/g) calculated from the adsorption-desorption N2 isotherms.

Sample	S_BET_	r_BET_	S_t_	r_t_
Mg/Al	109	0.9998	99	0.9999
Mg/Al/Fe	43	0.9998	43	0.9999
Mg/Fe	40	0.9997	40	0.9999
Mg/Al500	109	0.9984	108	0.9998
Mg/Al/Fe500	176	0.9997	175	0.9994
Mg/Fe500	104	0.9999	104	0.9992

**Table 6:** Kinetic study parameters for the kinetic models, pseudo-first order and pseudo-second order.

	Pseudo-First Order			Pseudo-Second Order		
	q_e_ (mg g^−1^)	k_1_ (min^−1^)	r^2^	q_e_ (mg g^−1^)	k_2_ (g mg^−1^ min^−1^)	r^2^
Mg/Al	608.50	0.0458	0.9844	384.62	6.4 × 10^−5^	0.9817
Mg/Al/Fe	550.54	0.0234	0.8711	---	---	---
Mg/Fe	531.07	0.0451	0.9781	526.32	3.8 × 10^−5^	0.9265
Mg/Al500	291.26	0.0284	0.9692	285.71	1.7 × 10^−4^	0.9233
Mg/Al/Fe500	145.98	0.0099	0.9068	---	---	---
Mg/Fe500	238.25	0.0407	0.9557	222.22	1.8 × 10^−4^	0.8335

## Data Availability

All data generated or analyzed during this study are included in this article.
